# Discovery of Novel Type II Bacteriocins Using a New High-Dimensional Bioinformatic Algorithm

**DOI:** 10.3389/fimmu.2020.01873

**Published:** 2020-09-03

**Authors:** Nannette Y. Yount, David C. Weaver, Jaime de Anda, Ernest Y. Lee, Michelle W. Lee, Gerard C. L. Wong, Michael R. Yeaman

**Affiliations:** ^1^Division of Infectious Diseases, Los Angeles County Harbor-UCLA Medical Center, Torrance, CA, United States; ^2^Division of Molecular Medicine, Los Angeles County Harbor-UCLA Medical Center, Torrance, CA, United States; ^3^Lundquist Institute for Biomedical Innovation at Harbor-UCLA Medical Center, Torrance, CA, United States; ^4^Department of Mathematics, University of California, Berkeley, Berkeley, CA, United States; ^5^Departments of Bioengineering, Chemistry and Biochemistry, University of California, Los Angeles, Los Angeles, CA, United States; ^6^The California NanoSystems Institute, University of California, Los Angeles, Los Angeles, CA, United States; ^7^Department of Medicine, David Geffen School of Medicine at UCLA, Los Angeles, CA, United States

**Keywords:** bacteriocin, host-defence, anti-infecive agents, computational biology, antimicrobial

## Abstract

Antimicrobial compounds first arose in prokaryotes by necessity for competitive self-defense. In this light, prokaryotes invented the first host defense peptides. Among the most well-characterized of these peptides are class II bacteriocins, ribosomally-synthesized polypeptides produced chiefly by Gram-positive bacteria. In the current study, a tensor search protocol—the BACIIα algorithm—was created to identify and classify bacteriocin sequences with high fidelity. The BACIIα algorithm integrates a consensus signature sequence, physicochemical and genomic pattern elements within a high-dimensional query tool to select for bacteriocin-like peptides. It accurately retrieved and distinguished virtually all families of known class II bacteriocins, with an 86% specificity. Further, the algorithm retrieved a large set of unforeseen, putative bacteriocin peptide sequences. A recently-developed machine-learning classifier predicted the vast majority of retrieved sequences to induce negative Gaussian curvature in target membranes, a hallmark of antimicrobial activity. Prototypic bacteriocin candidate sequences were synthesized and demonstrated potent antimicrobial efficacy *in vitro* against a broad spectrum of human pathogens. Therefore, the BACIIα algorithm expands the scope of prokaryotic host defense bacteriocins and enables an innovative bioinformatics discovery strategy. Understanding how prokaryotes have protected themselves against microbial threats over eons of time holds promise to discover novel anti-infective strategies to meet the challenge of modern antibiotic resistance.

## Introduction

One of the most urgent threats facing medicine and society today is the emergence of multi-drug resistant (MDR) pathogens. Estimates from the World Health Organization and like agencies suggest deaths due to MDR infections will outpace nearly all other causes by the year 2050 (1, 2). Compounding this issue is reduced pharmaceutical investment in anti-infective drug discovery, yielding a dearth of mechanistically novel anti-infectives in the drug development pipeline.

Virtually all modern anti-infectives identified to date were originally derived from microbial sources. Among these, bacteriocins are the earliest host defense peptides (HDPs), derived from bacteria to protect against microbial competitors. Although they originated in prokaryotes, HDPs have been retained throughout evolution and have been identified in virtually all organisms from which they have been sought. Such HDPs are typically small, cationic and amphipathic, and structurally categorized as predominantly α-helical, β-sheet or more complex secondary structure architecture, such as the cysteine-stabilized-αβ peptides. Mechanistically, a body of experimental data indicated that cationicity and amphipathicity as distributed in 3-dimensional space are essential for antimicrobial functions of HDPs. For example, cationicity is likely important for their propensity to target electronegative microbial membranes, while amphipathicity is likely essential for subsequent membrane perturbing events.

Bacteriocins are represented by a number of highly diverse families created through ribosomal or non-ribosomal synthesis (3–6). Of those generated by ribosomal synthesis, perhaps the best characterized are the Class II bacteriocins produced mainly by Gram-positive bacteria (7, 8). Class II bacteriocins are typically small (<60 amino acids) and heat-stable, and often synthesized as pre-bacteriocins containing an N-terminal signal sequence that is cleaved during secretion (4, 7, 8). This family of bioactive peptides can be further subclassified: Class IIa (pediocin-like); Class IIb (dimeric); Class IIc (cyclic) (4, 8). Hallmark of the Class IIa bacteriocins is an N-terminal consensus sequence (KYYGNG[L/V]XCXKXXCXVDW) comprised of an anti-parallel β-sheet stabilized by a disulfide bridge that is integral to antimicrobial activity (4, 7)

Previous investigations seeking to find novel bacteriocin sequences largely used computational screens for a conserved signal peptide motif (9, 10). However, in many of these investigations, this signal term has been class-specific, such that genomic screens that do not account for degeneracy, codon-use biases or open-reading-frame limitations are negatively restricted. Hence, while highly specific, such scans have missed large groups of bacteriocin sequences (10). In the present investigation, a novel and high-dimensional bioinformatics strategy—the BACIIα algorithm—was developed to overcome the above limitations. It incorporates a relaxed signal peptide motif that is inclusive of consensus bacteriocin leader sequences, along with key physicochemical and genomic pattern recognition to selectively identify putative bacteriocins from published sequence databases. Furthermore, this algorithm targets the α-helical core element of bacteriocins as a means to power specificity and sensitivity. Application of the novel BACIIα protocol retrieved all families of known Class IIa and IIb bacteriocin peptides, validating its inclusive scope. Moreover, it discovered >700 putative new bacteriocin sequences, many from prokaryotes for which no bacteriocin had been characterized to date. The retrieved sequences were predicted by a validated machine-learning classifier (11–14) with high probability to induce negative Gaussian curvature (NGC) in target membrane structures, which is a hallmark of antimicrobial activity. As proof-of-principle, prototype bacteriocin candidates were synthesized and found to have potent microbicidal activity against a panel of medically-relevant and drug resistant pathogens. Together, these data suggest the BACIIα algorithm is a rapid and efficient tool to identify novel bacteriocins which have retained efficacy against MDR pathogens over an evolutionary timespan. In this light, a greater understanding of host defense peptides that prokaryotes use to protect themselves against microbial competitors holds promise for discovery and development of innovative anti-infectives to meet the burgeoning threat of multi-drug resistant infections.

## Methods

### Generation of the Type II Bacteriocin Consensus Formula (BACIIα)

To identify a consensus formula inclusive of known class II bacteriocins, multiple sequence alignments integrating prototypic representatives of this family were carried out using CLUSTALW2 (https://www.ebi.ac.uk/Tools/msa/clustalw2/) and refined using MEGA 6 (15). Sites of potential conservation were scored for residue or physicochemical identity to generate a 12-residue core consensus formula. In some cases, positions in the formula are degenerate for inclusivity, based on sequence or biochemical (polar residues) properties conserved at these positions. Initial sequence alignments were generated using CLUSTALW2, followed by manual adjustment to align the double glycine motif using MEGA 6 (15).

### Screen for Amphipathic α-Helices Within Retrieved Dataset

This consensus formula, termed BACII**α**, was then used with ScanProsite (https://prosite.expasy.org/scan-prosite/) to conduct computational pattern searches of the UniProtKB Swiss-Prot and TrEMBL databases (https://www.uniprot.org/). Search results were further filtered for: (1) protein size (<80 residues); (2) bacterial source; and (3) localized to the first 25 residues of the query protein with a “ < X(0.25)” logical operator. Results were submitted as a sequence database against which additional pattern searches could be carried out using Prosite. This database was queried using a systematic degenerate amphipathic sequence formula strategy [(11); https://prosite.expasy.org/scan-prosite/] to scan for α-helical domains within the retrieved protein dataset. The formula was advanced sequentially one position at a time through 18 iterations to encompass an entire 18-residue helical wheel span. Iteration one of this query sequence is listed below:

X-[VILMCFWYAG]-[KRHEDNQSTAG]-[KRHEDNQSTAG]-[VILMCFWYAG]-[VILMCFWYAG]-[KRHEDNQSTAG]-[KRHEDNQSTAG]-[VILMCFWYAG]-X-[KRHEDNQSTAG]-[VILMCFWYAG]

As mature bacteriocins are typically located near C-termini of holoproteins, search parameters included a “X(0.30)>” logical operator to restrict results to the final 30 residues of target proteins.

### Physicochemical Parameter Determination

Retrieved datasets were subjected to batch analysis to compute physicochemical parameters. The isoelectric point (pI) of individual sequences was determined using ExPASy (https://web.expasy.org/compute_pi/), while the hydrophobic moment, mean hydrophobicity, net charge (K and R [+1], H [+0.5], D and E [−1]) and K and R residue frequency (*N*_*K*_*/N*_*K*_+*N*_*R*_) were determined using Python programs coded for this purpose. Residue frequency analysis was carried out using the Sequence Manipulation Suite in Protein Stats (https://www.bioinformatics.org/sms2/).

### Genomic Operon Characterization

To probe for unforeseen or novel bacteriocins, genomic regions surrounding uncharacterized hits were analyzed. A total of 20,000 base pairs (10,000 each upstream and downstream) from search hit sequences were scored for the presence of typical bacteriocin operon genes (e.g., ABC transporters, immunity proteins, pheromones). Sequences consistent with bacteriocin-operon genomics signatures were prioritized for further study.

### Design of the BACIIα Algorithm

Multiple structural elements may impact antimicrobial activity of host defense peptides, including biochemical features such as sequence motifs and electrostatic charge. However, of key importance to overall antimicrobial function is how these physicochemical properties are distributed in 3-dimensional space. To improve specificity and probe for membrane-active amphipathic α-helical structures which are important for membrane permeabilization and antibacterial activity, sequences retrieved from 1° searches were subjected to further computational screens collectively comprising the BACIIα algorithm:

#### Amphipathic Helix Motif

The BACIIα sequence formula returns hits based on their sequence alignment. To assess 3-dimensional patterns, hit sequences were assessed using a recently-identified tool that identifies core signatures of α-helical antimicrobial peptides [termed the α-core; (11)]. This analysis enabled spatial patterns of residues encompassed in the helical domains of retrieved proteins.

#### Physicochemical Profile

Proteins were also scored for intrinsic physicochemical parameters including: electrostatic charge [Q]; hydrophobic moment [μH]; mean hydrophobicity [H]; isoelectric point [pI]; and lysine-to-arginine ratio (*N*_*K*_*/N*_*K*_+*N*_*R*_). These analyses were performed using Python algorithms specifically created for this study. Hydrophobicity values were derived using the Fauchère and Pliska octanol-water interface scale (16). PI was calculated using the ExPASy Compute pI/MW tool https://web.expasy.org/compute_pi/).

### Machine-Learning Validation

To further characterize the datasets retrieved by the BACIIα formula, a previously developed support vector machine (SVM)-based classifier (12–14) was used to screen the obtained sequences for antimicrobial activity. Briefly, the SVM classifier was trained to optimally partition known α-helical sequences present in the Antimicrobial Peptide Database [APD, http://aps.unmc.edu/AP/main.php; (17)] from decoy peptides with no reported antimicrobial activity. The SVM generated 12 descriptors from the peptide sequence and output a score σ specifying the distance of the peptide from the 11-dimensional hyperplane separating antimicrobial and non-antimicrobial sequences. Using small-angle X-ray scattering (SAXS) experiments, the σ scores were found to correlate with the ability to generate NGC by α-helical test sequences (12). Thus, a large, positive σ score correlates with the ability to induce NGC in membranes, whereas a negative σ score indicates a lack of NGC activity. This membrane curvature feature is characteristic of antimicrobial peptides that have cell membrane-permeating functions (12–14). Sequences retrieved from the α-core search tool were screened using this algorithm, and σ scores calculated. Spearman correlations were quantified between σ and α-core metrics using Mathematica software (https://www.wolfram.com/mathematica/online/).

### Synthesis of Prototypic Bacteriocin Candidates

Select putative bacteriocin sequences were commercially synthesized (BioMatik, https://www.biomatik.com/) at a 100 mg scale. All sequences were authenticated for mass and amino acid composition and purified using RP-HPLC to >98% purity. Lyophilized peptides were reconstituted with double-distilled and deionized water (ddIH20) and stored in aliquots at −20°C.

### Antimicrobial Assay

Antimicrobial assays of putative bacteriocins were performed using a well-established radial diffusion method at pH 5.5 (a surrogate for contexts of serum or acidic phagolysosomes) or 7.5 [a surrogate for bloodstream context; (18)]. These peptides were assayed for antimicrobial activity against a panel of human pathogens paired for susceptibility (S) or resistance (R) phenotypes: Gram-positive *Staphylococcus aureus* [ISP479C [S], ISP479R [R]; (19)]; Gram-negative *Salmonella typhimurium* [MS5996s [S], MS14028; (20)], *Pseudomonas aeruginosa* (PA01 [R]), *Acinetobacter baumanni* (17,928; [R]) and the fungus *Candida albicans* [36082S [S] or 36082R [R]; (21)]. In brief, logarithmic-phase organisms were inoculated (10^6^ CFU/ml) into buffered agarose, and poured into plates. Peptides (10 μg) were introduced into wells in the seeded matrix, and incubated for 3 h at 37 °C. Nutrient overlay medium was applied and assays incubated at 37 or 30°C for bacteria or fungi, respectively. Zones of inhibition were quantified after 24 h incubation. Independent experiments were repeated a minimum of three times, and assessed by parametric analysis for statistical significance (22).

## Results

### Defining the BACIIα Probe Sequence Formula

**Figure 1:**
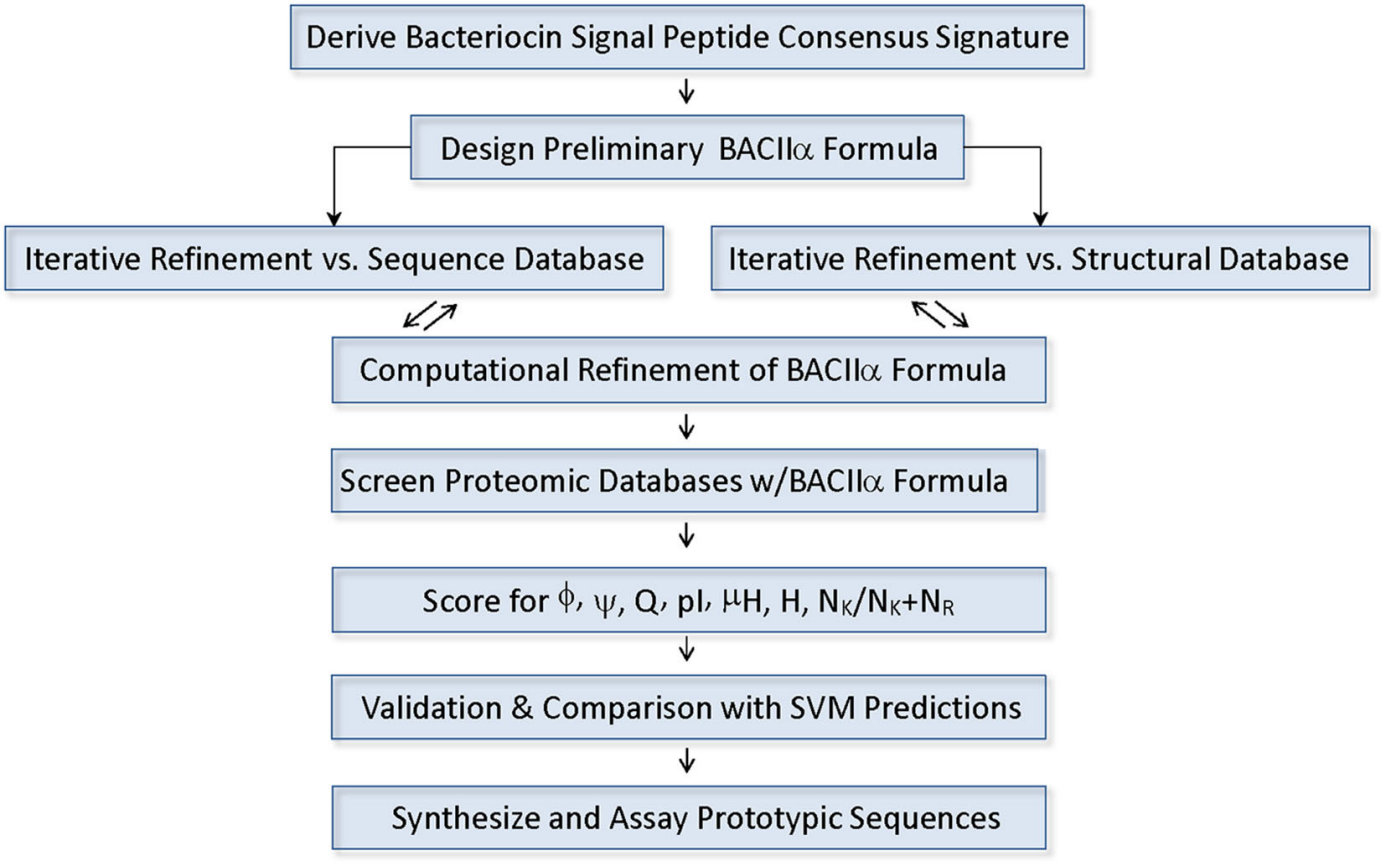
Components and Process of the BACIIα Algorithm.

A consensus formula consistent with the vast majority of known Class II (a-d) bacteriocins was identified and used to probe protein databased (Figure 1). Conserved residues in the signal peptide domain were used to generate a 12 residue consensus element comprising the formula:

$$\frac{\begin{array}{lllll} -12 & \;-11 & \;-10 & \;-9 & \;-8\;-7\;-6\;-5\;-\;4\;-3\;-2\;-1 \end{array}}{[\text{LI}]-[\text{KREDNQSTYH}]-\text{X}-[\text{KREDNQSTYH}]-\text{X}-[\text{MLV}]-\text{X}-\text{X}-[\text{IVLT}]-\text{X}-\text{G}-\text{G}}$$

Notably, several positions within this formula were conserved predominantly at the level of physicochemical properties (positions −9 and −11). These positions are represented by degenerate search terms reflecting the propensity for a polar residue at these positions. Using this BACIIα probe sequence formula, a primary computational pattern search of the UniProtKB/Swiss-Prot and TrEMBL databases yielded a total of 3,050 sequences. Of the characterized sequences (706), the following bacteriocin-related classes were represented: bacteriocins (53%); competence enhancing peptides (18%); auto-inducing peptides (2%); and pheromones (1%) (Table 1).

**Table 1:** Retrieved sequences using BACIIα algorithm by stage of study.

**Group**	**Signal peptide search**	**%**	**Amphipathic pattern search**	**%**
**Characterized sequences**				
Bacteriocins	376	53	308	82
Competence enhancing peptides	129	18	6	2
Pheromones	7	1	1	0.3
Autoinducing peptides	12	2	8	2
Other	182	26	52	14
Total characterized Sequences	706	–	375	–

Collectively, 74% of known characterized sequences were bacteriocin or bacteriocin-related sequences.

### Application of the BACIIα Algorithm

Applying the BACIIα algorithm, the total number of high-priority sequences was 1,563. Among the characterized sequences (375), 82% were bacteriocins and 4% included other bacteriocin-related sequences (Table 1). Hence, application of the BACIIα algorithm increased specificity for bacteriocins from 53 to 82%. Inclusion of bacteriocin-related peptides increased specificity to 86% within the subset of proteins having known functions. The resulting dataset included members from nearly all Class IIa and IIb bacteriocin families within the UniProtKb database (Table 2). In particular, the formula identified representatives from ~90% of Class IIa families and 88% of Class IIb families. Class IId (other) structural class bacteriocins were less predominant (13%). As expected, representatives from the cyclic, Class IIc bacteriocin group, which do not contain a helical element, were not retrieved with this search. For many bacteriocins more than one representative of each family was retrieved; and in some cases a large number of family members were returned, such as for the Class IIb Lactobin family where more than 70 members were identified.

**Table 2:** Bacteriocin peptides retrieved by multi-component formula search.

**Class**	**Peptide**		**Organism**
IIa	Acidocin Avicin Carnobacteriocin Curvacin Divergicin Enterocin Leucocin Mundticin PapA Pediocin Piscicolin Plantaricin Sakacin	8912, LF221B, M A A, B2, BM1 A 750 B, 1071A/1B, CRL35, C2, NKR-5-3 A, A-Qu 15, B, C, K, N, Q KS, L PA-1, AcH 126 A, F, J, 1.25 beta, NC8, c81F, S A, D98c, P, T, X	*Lactobacillus acidophilus* *Enterococcus avium (Streptococcus avium)* *Carnobacterium maltaromaticum* *Lactobacillus curvatus* *Carnobacterium divergens (Lactobacillus divergens)* *Enterococcus faecium* *Leuconostoc gelidum, Leconostoc carnosum,* *Enterococcus pallens ATCC BAA-351* *Listeria aquatica FSL S10-1188* *Pediococcus acidilactici* *Carnobacterium maltaromaticum* *Lactobacillus plantarum* *Lactobacillus sakei*
IIb	Amylovorin Bacteriocin Brevicin Gassericin Lactobin/Cerein Lactocin Lactacin F	L alpha, L beta, L471 GatX, BacSJ2-8 925A T A/7B 705 alpha, 705 beta LafA, LafX	*Lactobacillus amylovorus* *Streptococcus pneumoniae* *Lactobacillus brevis* *Lactobacillus gasseri* *Streptococcus australis ATCC 700641* *Lactobacillus curvatus* *Lactobacillus johnsonii*
IId	Lactococcin Mesentericin Weissellicin	A, A1, G beta, Q beta B105, Y105 L	*Clostridium perfringens (strain SM101 / Type A)* *Leuconostoc mesenteroides* *Weissella hellenica*

### Origin Species Classification

The majority of sequences (bacteriocins and related) retrieved with the BACIIα formula originated from Gram-positive Firmicutes (74% [50% *Lactobacillus* spp.; 14% other *Bacillus* spp.; 10% *Clostridium* spp.]) and other Gram-positive organisms (Actinobacteria [2%]). Sequences were also retrieved from a number of Gram-negative organisms (Table 3). Additionally, a number of putative bacteriocins were retrieved from organisms for which bacteriocins have yet to be characterized.

**Table 3:** Source organisms of retrieved dataset proteins.

**Organism**	**Phylum**	**% Class**	**Class**	**% Subclass**
Gram-Positive				
	*Firmicutes*	74		
			*Lactobacilli*	50
			Other bacilli	14
			*Clostridiae*	10
	Other	2		
			*Actinobacteria*	2
Gram-Negative	*Proteobacteria*	24		
			*Bacteroides*	13
			*Proteobacteria*	9
			*Cyanobacteria*	2
			*Chlamydiae*	<1.0
			*Planctomycetia*	<1.0
Non-gram staining		<1.0		
			*Mollicutes*	<1.0
Unclassified		<1.0		<1.0

### Physicochemical Properties of Known Bacteriocins

**Figure 2:**
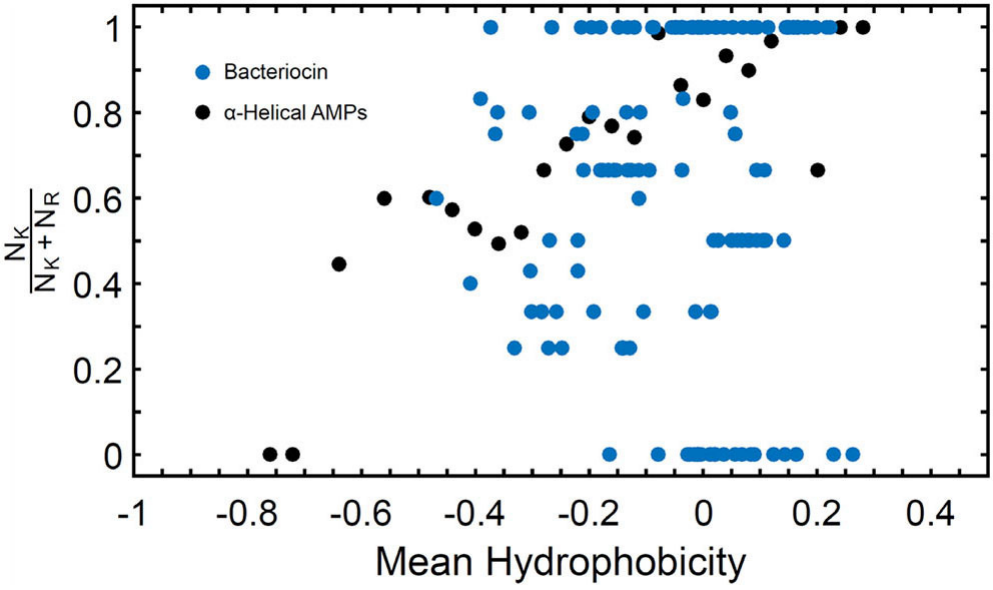
N_K_/N_K_+N_R_ Ratio and Mean Hydrophobicity in Study Molecules. Percentage of lysine (N_K_) relative to arginine (N_R_) expressed as (N_K_/N_K_+N_R_) vs. hydrophobicity (H) in study αHDPs Preference of lysine as compared to arginine is reflected in an increased value of H for peptides capable of generating NGC in membranes as predicted by the saddle-splay rule.

Known bacteriocins retrieved using the BACIIα formula were analyzed for multiple physicochemical properties. The amphipathic spans of the 308 identified bacteriocins had the following average values: charge (Q), +1.1; hydrophobic moment (μH), 0.33; and mean hydrophobicity (H), 0.46 (Table 4). The lysine to arginine ratio (*N*_*K*_*/N*_*K*_+*N*_*R*_) indicated lysine was preferred over arginine at an ~2:1 ratio, particularly at positions 1, 8 and 15, nearer the termini of helices. Moreover, as the *N*_*K*_*/N*_*K*_+*N*_*R*_ ratio increased over amphipathic spans, so did net hydrophobicity (Figure 2). This finding suggests that lysine propensity is compensated by increasing hydrophobicity in bacteriocins or other HDPs (11, 23).

**Table 4:** Biophysical properties of retrieved dataset proteins.

**Group**	***n***	**%**	**μH**	**Q**	**N_K_/N_K_+N_R_**	**H**	**PI**
Known bacteriocins	308	22	0.33 (±0.2)	1.1 (±1.5)	0.71 (±0.3)	0.46 (±0.1)	6.8 (±2.3)
Bacteriocin-related^*^	15	1	0.51 (±0.1)	1.9 (±1.9)	0.85 (±0.9)	0.37 (±0.2)	8.5 (±2.3)
Non-bacteriocin	52	4	0.40 (±0.1)	0.4 (±1.5)	0.42 (±0.4)	0.43 (±0.2)	7.1 (±2.4)
Uncharacterized	1038	73	0.39 (±0.2)	0.1 (±1.5)	0.68 (±0.4)	0.41 (±0.4)	6.4 (±2.1)

* *Includes pheromones, competence-inducing peptides and others*.

*μH, hydrophobic moment; Q, charge; NK/NK+NR relative percentage of lysine vs. arginine; H, hydrophobicity; PI, isoelectric point. Values are presented ± standard deviation*.

### Global Residue Frequencies

**Figure 3:**
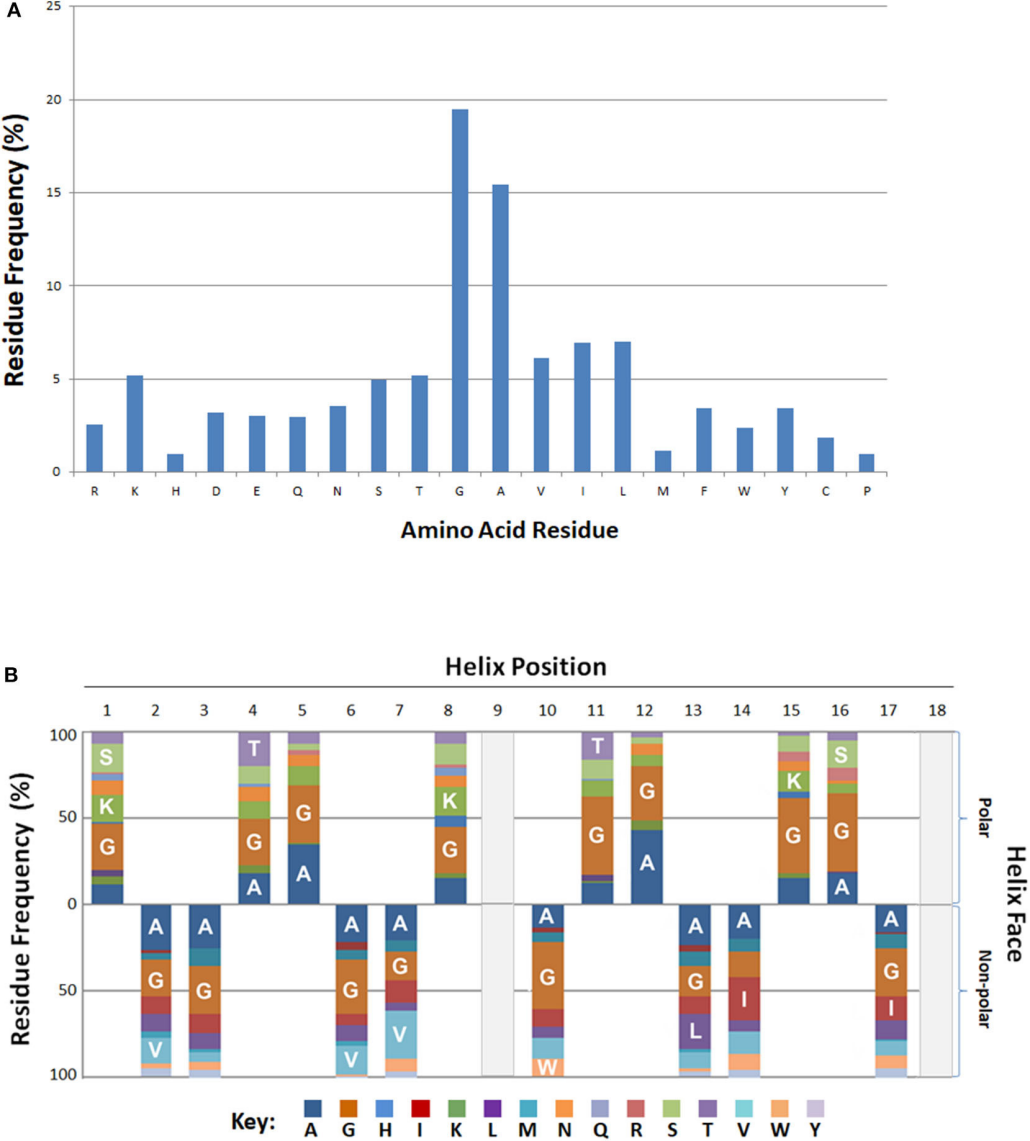
Positional and Spatial Amphipathic Residue Frequency. **(A)** Relative amino acid percentages are displayed for bacteriocins. **(B)** Percentages of individual residues associated with either the polar or non-polar search term group are represented as various color blocks. Residues above the x-axis are associated with the polar residue group and residues below the axis are found on the.

Residue frequency analyses of known bacteriocins revealed an enrichment in certain residues. In particular, residues glycine and alanine collectively represented more than one third (35%) of all amino acids (Figure 3A). Of the charged residues, the basic amino acid lysine (5%) was the most abundant. Other cationic (R) and anionic (D, E) residues were represented at a lower frequency overall (~3%). The aliphatic (non-polar) hydrophobes, leucine, isoleucine or valine had equivalent frequencies (6–7%), and occurred nearly twice as often as the aromatic hydrophobes phenylalanine, tryptophan or tyrosine (2.4–3.5%).

### Positional and Spatial Residue Frequencies

The BACIIα formula identifies hits based on alignment to its sequence formula. Three-dimensional assessment is also informative regarding positional and spatial localization of residues along the identified amphipathic spans (Figure 3B). Glycine and alanine, the most abundant residues, were distributed across the amphipathic spans and found on both hydrophobic and hydrophilic facets with a similar frequency. On the polar facet, the next most abundant residues were the cationic residue lysine and neutral hydrophilic residues threonine and serine. On the non-polar facet, the most abundant residues were the aliphatic hydrophobes, valine, leucine and isoleucine.

### Analysis of Uncharacterized Sequences

Beyond retrieving known bacteriocins, the BACIIα algorithm identified a large number (1,038) of as yet uncharacterized sequences. To assess this sequence dataset based on physicochemial properties of known bacteriocins, we applied a mathematical scoring system of factors inherent to membrane permeabilizing, microbicidal sequences (11). Hydrophobic moment (μH) and net charge (Q), represented by a combinatorial index μH^*^Q (HMQ), were quantified. These data were binned and values representing the top 25th and 50th highest HMQ quartiles (HMQ_25_ and HMQ_50_) were derived. Application of these thresholds revealed a significant portion of the uncharacterized dataset (*n* = 208, HMQ_25_; *n* = 319, HMQ_50_) are likely to have antimicrobial properties (Table 5). Therefore, more than 700 (>74%) of the uncharacterized molecules retrieved by the BACIIα algorithm are putative novel bacteriocins.

**Table 5:** Quartile analysis of dataset protein properties vs. SVM scoring.

	**Original *n***	**Subset *n***	**%**	**μH**	**Q**	**N_K_/N_K_+N_R_**	**H**	**PI**	**σ**
Category	Total	μH*Q > 1.0							SVM
Known bacteriocins	308	43	14	0.52	3.2	0.68	0.38	8.68	0.90
Bacteriocin-related	15	9	60	0.56	3.4	0.91	0.29	9.98	0.64
Non-bacteriocin	52	10	19	0.52	2.7	0.27	0.34	8.18	0.72
Uncharacterized	1,038	85	8	0.53	3.2	0.56	0.33	8.67	0.91
Category	Total	μH*Q > 0.50							SVM
Known bacteriocins	308	79	26	0.46	2.6	0.69	0.42	7.9	0.80
Bacteriocin-related	15	10	66	0.57	3.2	0.92	0.32	9.6	0.63
Non-bacteriocin	52	15	29	0.50	2.4	0.38	0.35	8.1	0.65
Uncharacterized	1,038	208	20	0.49	2.3	0.63	0.36	7.6	0.80
Category	Total	μH*Q > 0.25							SVM
Bacteriocins	308	161	52	0.36	2.1	0.75	0.42	7.2	0.58
Bacteriocin-related	15	12	80	0.52	2.8	0.76	0.35	9.1	0.54
Non-bacteriocin	52	16	31	0.50	2.3	0.4	0.35	7.9	0.67
Uncharacterized	1,038	319	31	0.44	1.9	0.65	0.38	7.2	0.65

The μH*Q values represent different percentile cutoffs for peptide groups (dark orange, >1.0; middle orange; >0.50; and light orange, >0.25). Definition legend: μH–hydrophobic moment; Q–charge; N_K_/N_K_+N_R_ relative percentage of lysine vs. arginine; H–hydrophobicity; PI–isoelectric point.

### Membrane Active Propensity

**Figure 4:**
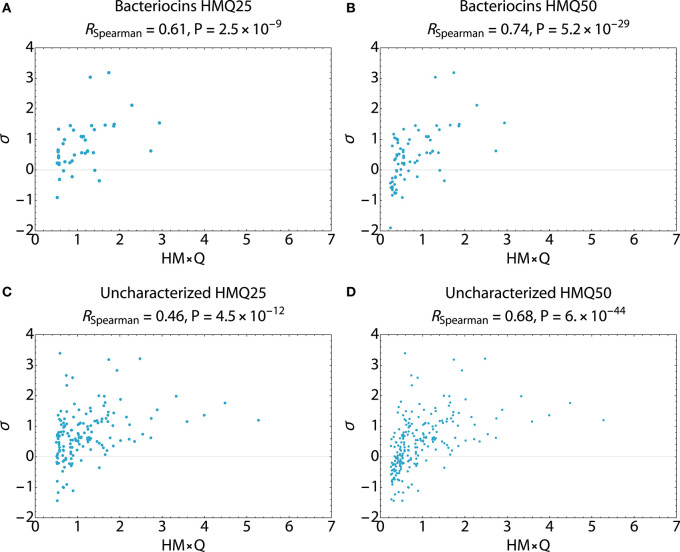
Spearman Correlations Multi-Component BACIIα Formula and ML Classifier. Correlations were carried out to assess the predictive accuracy and monotonic ranking between the BACIα algorithm and the SVM classifier scored peptide sequences. Plots compare HMQ (BACIIα predictive) vs. sigma (classifier probability) scores for study peptides in the top 25th (HMQ25) and 50th (HMQ50) percentiles. The bacteriocin groups **(A,B)** display scores for identified bacteriocins. The uncharacterized groups **(C,D)** reflect those peptides which are also predicted to be membrane permeabilizing by the two protocols. All comparisons were found to be significant given a cutoff value of *P* ≤ 0.05. Correlations were carried out using Mathematica (Wolfram).

Search hits were assessed for membrane active propensity characteristic of antimicrobial peptides (Table 5). The sequence dataset was evaluated using a validated SVM machine-learning classifier for sequences capable of generating negative Gaussian curvature in model membranes (12–14). The SVM algorithm integrates specific physicochemical parameters such as amphipathicity (μH), charge (Q), and sequence-order. The output score, σ, quantifies confidence of this classification; high positive σ values have high probability of NGC which is characteristic of membrane permeabilizing, antimicrobial properties. The known bacteriocins retrieved were predicted to be membrane active, with average σ scores of 0.80 (HMQ_25_) and 0.58 (HMQ_50_). Likewise, a high percentage of the dataset encompassing unknown proteins was also predicted to have membrane permeabilizing activities with σ scores of 0.80 (HMQ_25_) and 0.65 (HMQ_50_). To test the accuracy of the BACIIα retrieved datasets relative to the SVM classifier, Spearman correlations were performed to assess monotonic ranking. This assessment revealed highly significant correlations (*R* = 0.46–0.74; range, *P* = 2.5 × 10^−9^ to 6.0 × 10^−44^) between datasets generated by the two methods (Figure 4). This strong congruence suggests the BACIIα algorithm accurately detects unforeseen antimicrobial sequences (e.g., novel bacteriocins) and converges with the SVM on attributes conferring microbicidal properties.

### Selection of Bacteriocin Candidates

**Figure 5:**
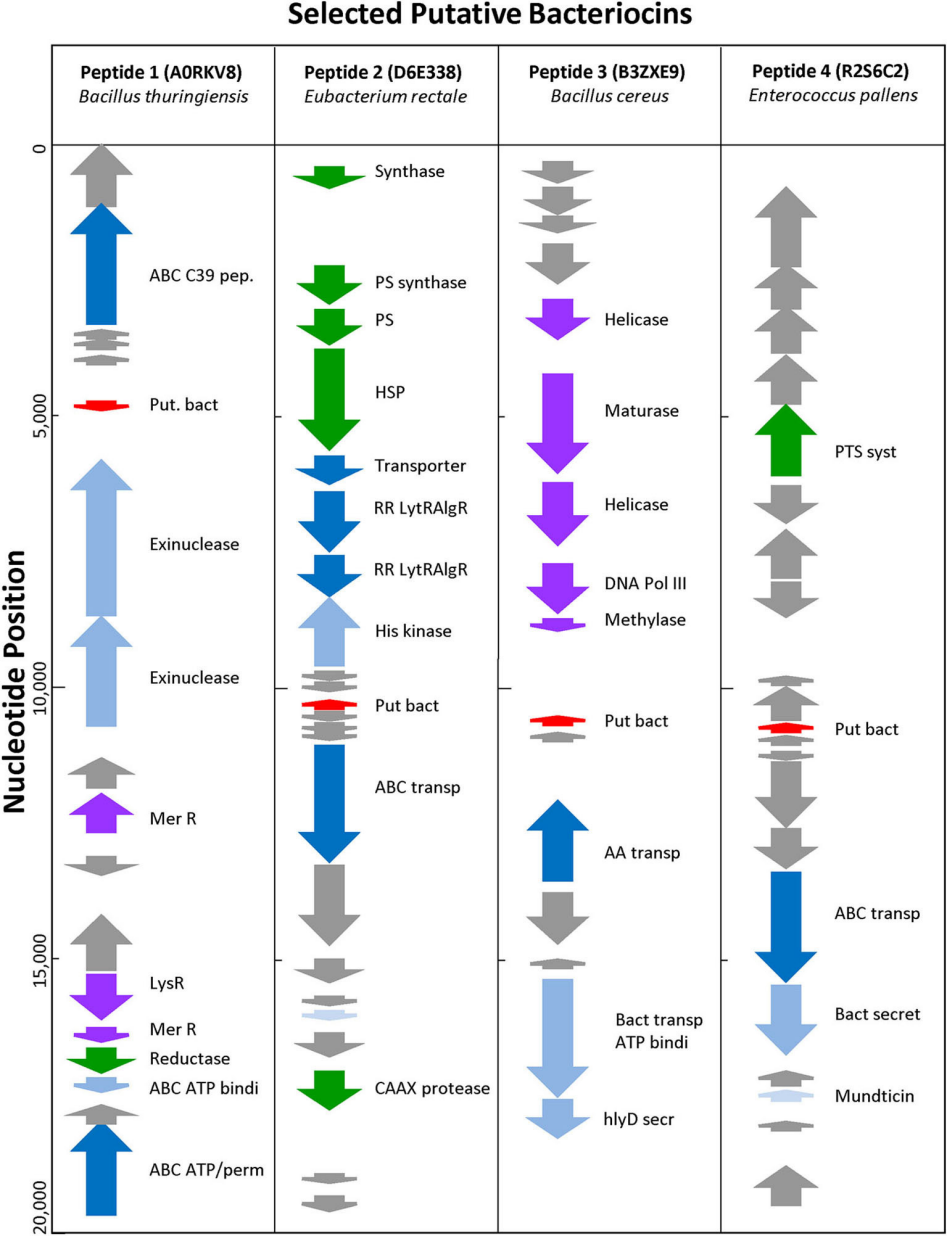
Genomic Environment Surrounding Putative Bacteriocins. Analysis of 20 kb region surrounding putative bacteriocin genes. Red—putative bacteriocin; gray—hypothetical proteins; dark blue—C39 bacteriocin processing peptidase; medium blue—exinuclease ABC subunit; light blue—ABC transporter, ATP binding protein; green other enzyme; purple—polymerase related protein.

Uncharacterized sequences representing putative novel bacteriocins were selected based on high BACIIα algorithm scores and genomic analyses. Among these, sequences from phylogenetically distinct organisms were chosen to assess correlates of source and target organisms: (SwissProt accession [*species*; study name]): A0RKV8 (*Bacillus thuringiensis*; peptide-1); D6E338 (*Eubacterium rectale*; peptide-2); B3ZXE9 (*Bacillus cereus*; peptide-3); R2S6C2 (*Enterococcus pallens*; peptide-4). At a genome level, peptides 1–4 localized to bacteriocin-like operons containing bacteriocin-associated genes (Figure 5). All were localized within 20 kb of an ABC transporter protein and ABC transporter accessory genes, such as C39 peptidases and ATP binding proteins. Candidate bacteriocins also localized within gene loci characteristic of known bacteriocin sequences and/or pheromones. In some cases, prototypic bacteriocin immunity peptides also localized to putative bacteriocin operons.

### Antimicrobial Activity of Bacteriocin Candidates

**Figure 6:**
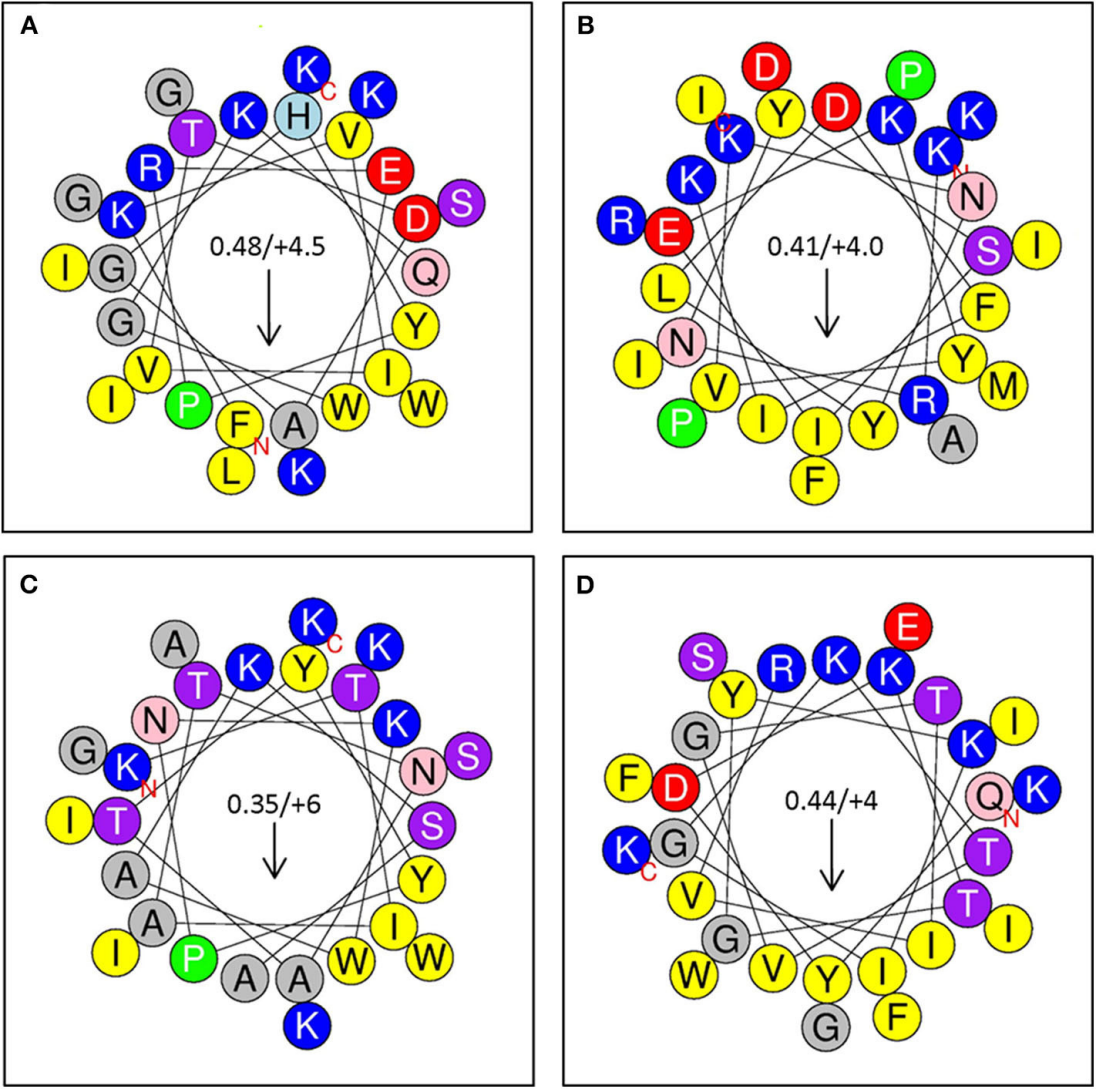
**(A)** Sequence Analysis and Antimicrobial Activity of Putative Bacteriocins. Putative bacteriocins synthesized for assessment of antimicrobial activity. Arrows indicate hydrophobic moment and direction. **(A)** Peptide 1: A0RKV8 (+4.5), PI−10.7; *Bacillus thuringiensis* (G+); FKVIVTDAGHYPREWGKQLGKWIGSKIK (24); **(B)** Peptide 2: D6E338 (+4), PI 10.3; *Eubacterium rectale;* KRNYSIEKYVKNYlDFIKKAIDIFRPMPI (25); **(C)**; Peptide 3: B3ZXE9 (+6), PI−10.9; *Bacillus cereus*; KTIATNATYYPNKWAKSAGKWIASKIK (26). **(D)** Peptide 4: R2S6C2 (+4), PI−10.5; *Enterococcus pallens*, QYDKTGYKIGKTVGTIVRKGFEIWSIFK (24).

**Figure 7:**
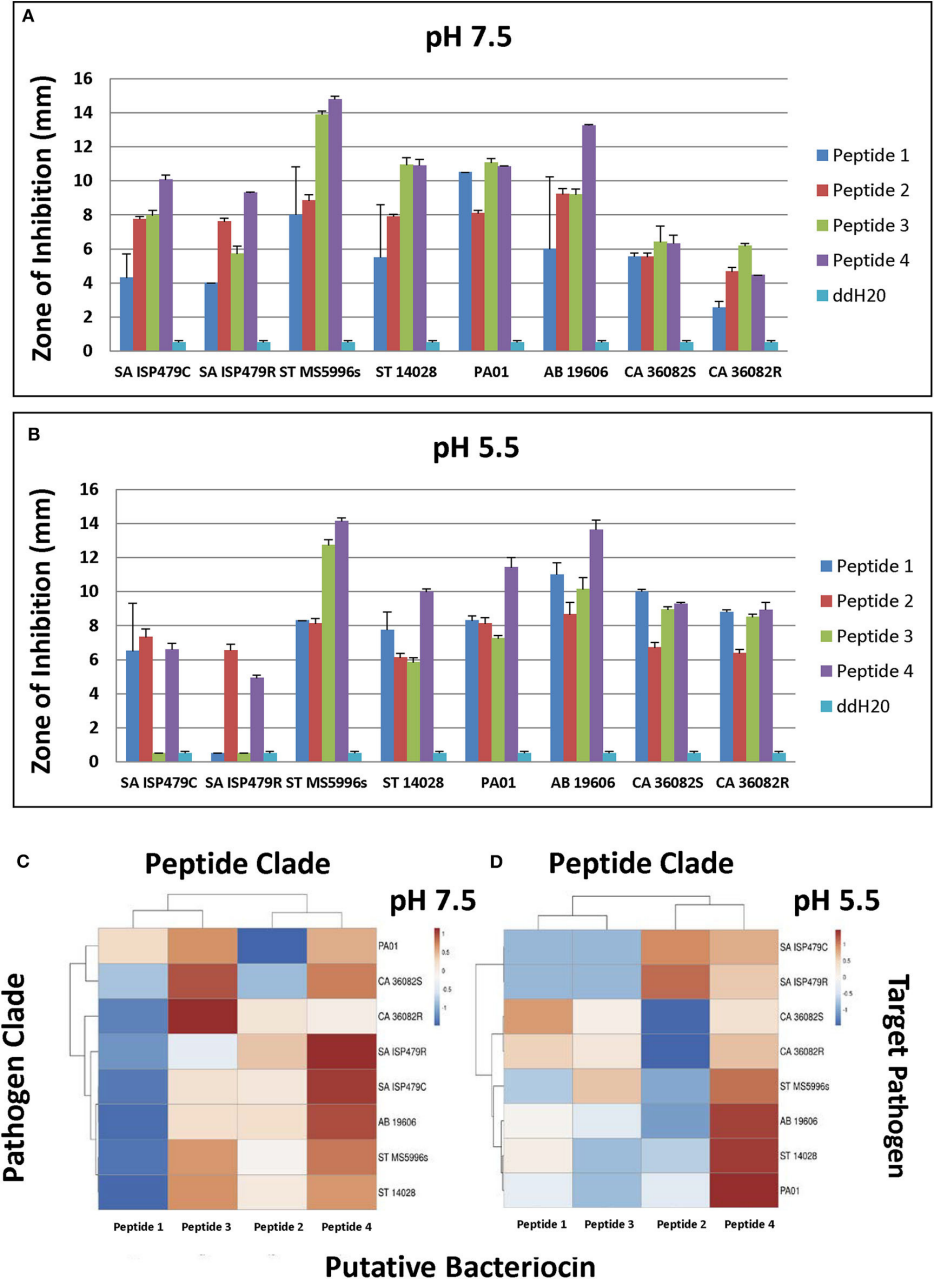
Microbicidal activity of study test peptides vs. a panel of prototypic gram-positive (*S. aureus*), gram-negative (*S. typhimurium, P. aeruginosa, A. baumannii*) and fungal (*C. albicans*) pathogens at two pH representing: **(A)**–bloodstream (pH 7.5); or **(B)**–phagolysosomal/abscess (pH 5.5). Data represent experiments independently performed a minimum of *n* = 3 times. Error bars represent the standard error of the mean. All study peptides were found to have statistically significantly greater activity (*P* < 0.01) than the dilution vehicle (ddH_2_0) in at least one pH condition. Note the differential pH dependent efficacy of Peptide 3 against *S. aureus*. The relative efficacies of study peptides against representative organisms at pH 7.5 or pH 5.5 are shown in the cluster analyses in panels **(C,D)**, respectively (red, relatively greater efficacy; blue, relatively lesser efficacy).

Selected peptides 1–4 (Figure 6) were assessed for antimicrobial activity against a panel of human pathogens (Figures 7A,B). All four putative bacteriocins possessed microbicidal activity against Gram-positive (*S. aureus*), Gram-negative (*S. typhimurium, P. aeruginosa, A. baumanni*) and a fungus (*C. albicans*). While active against all organisms tested, peptides 1–4 had generally greater activity vs. Gram-negative pathogens. The relative activity of peptides 1–4 was greater at pH 7.5 than at pH 5.5. Notably, peptide three lost nearly all activity against the Gram-positive pathogen *S. aureus* at pH 5.5. Beyond individual efficacy, cluster analyses reveal patterns of peptide efficacy against organism groups and as influenced by pH. For example, at pH 7.5, peptide one was relatively less active than the other peptides against all organisms except *Ps. aeruginosa* (Figures 7C,D).

## Discussion

Class II bacteriocins are typically small, cationic peptides of bacterial origin that often contain a conserved signal sequence important for downstream processing of the mature peptide. This leader domain is characterized as having a highly conserved double-glycine motif essential for proper cleavage of the bacteriocin precursor and maturation of the active mature peptide (4, 6, 27). Prior reports have made use of the signal peptide consensus to search for unidentified bacteriocin sequences in published genomic or proteomic sequence databases (28). However, these studies largely employed a very strict formulae [e.g., LSX_2_ELX_2_IXGG; (29)], often selecting only the most abundant residue at a position as a component of their search term. Hence, results conveyed a high degree of specificity, but had very limited sensitivity to identify novel bacteriocin molecules or classes within emerging proteomic databases.

In the present study, an alignment of more than 200 prototypic class II bacteriocins was carried out to generate an inclusive consensus formula. A primary component of this BACIIα formula was a convergent signal sequence. In addition to the C-terminal double glycine motif in this signal domain, the consensus formula included a strategic design to account for specific residues in key positions. For example, it allowed for inclusion of any polar residue at positions −9 and −11 of the signal peptide backbone. Further, a specific set of hydrophobic residues was allowed at positions −4 and −7. These features encompass the class II bacteriocin leader consensus originally identified by Nes and colleagues (6). The resulting consensus signature formula, BACIIα, represents an innovative probe for unforeseen bacteriocins. This formula retrieved members from nearly all known classes of type II bacteriocins, and the vast majority (~90%) of Class IIa and IIb linear bacteriocins.

The BACIIα formula was used as the first step in the multifactorial BACIIα search algorithm designed to discover novel bacteriocins. To improve specificity for membrane-active sequences characteristic of antimicrobial activity, the BACIIα algorithm integrated a strategy to probe for α-helical domains in retrieved peptides (11). The current results are in concordance with Class IIa and IIb bacteriocin propensity to adopt α-helical conformation in membrane mimetic environments (4). One notable exception was for the Class IIa pediocins, which were retrieved by the BACIIα formula, but not with the α-helix screen. This result would be expected, as many members of the pediocin-like bacteriocin group form a hairpin-like structure at the C-terminus (26). Given the high efficiency and specificity with which it captures bacteriocins, the BACIIα sequence formula and ensuing BACIIα algorithm provide a comprehensive strategy to reveal previously unrecognized bacteriocins. For example, the BACIIα search algorithm discovered putative bacteriocin sequences that were not returned using existing bacteriocin identification tools (e.g., BAGEL3; data not shown). As BAGEL3 employs an internal ORF calling component, its limits may reflect a difficulty of identifying the very small ORFs (≤ 0.5 kb) that are typical of bacteriocins (24).

To support results of the BACIIα algorithm, retrieved bacteriocin candidates were analyzed using a validated SVM-learning classifier to score membrane-active propensity (12–14). The SVM analyses confirmed that the vast majority of proteins prioritized by the BACIIα algorithm were likely to have a propensity for generating NGC in membrane environments and be antimicrobial in nature. This congruence was supported by regression analyses that yielded robust statistical significance. Thus, the BACIIα and SVM protocols, which derive from highly divergent knowledge-based and machine-learning strategies, converge on the same set of bacteriocin candidates. As the SVM was previously shown to generate high σ values for eukaryotic HDPs, the current findings further suggest that core features integral to antimicrobial activity are conserved in HDPs from eukaryotic and prokaryotic hosts.

Residue frequency analysis of the BACIIα dataset revealed that alanine and glycine are strongly preferred among amphipathic spans in bacteriocins (>33% of residues). These residues are distributed to both the polar and non-polar facets in these proteins. Such findings lend support to a new hypothesis regarding the mechanism by which α-helical HDPs may limit self-toxicity. Specifically, an abundance of small, sterically-unrestrained residues with a high degree of rotational freedom (e.g., glycine and alanine) may serve to keep α-helical antimicrobial peptides in an unstructured and thus non-toxic conformation in aqueous environments. Only when adopting their amphipathic structure in context of the hydrophobic milieu of a target membrane do they become cytotoxic. The fact that HDPs typically have higher affinity for prokaryotic vs. eukaryotic membrane constituents enhances this antimicrobial specificity. Support for this hypothesis is provided by: (1) the abundance of glycine, and to a lesser extent alanine, in α-helical HDPs of many organisms (11); (2) structural studies (25, 30–32) finding that α-helical HDPs are often unstructured in aqueous solutions, and only adopt α-helical conformation in membrane environments; and (3) propensity for α-helical HDPs to target cardiolipin or phosphatidylglycerol moieties common to prokaryotic membranes, with less affinity for phospholipids or sterols more common to eukaryotic membranes. In the current study, the abundance of glycine and alanine in retrieved sequences suggests these peptides may also utilize a similar mechanism to limit self-toxicity. Prokaryotes also express other safeguards to protect themselves from the very bacteriocins they produce. For example, organisms which make bacteriocins also produce immunity proteins, encoded within the bacteriocin-producing operon, which help to minimize self-toxicity (4, 8). In this respect, bacteriocins made by one bacterium can preferentially kill other competitive or pathogenic bacteria or fungi. Therefore, bacteriocins have a plausible role in host defense against infection, be it the bacterium producing the bacteriocin, or the host in which it resides. These concepts form a fundamental tenet for the protective roles of the beneficial human microbiome (33, 34).

It was also of interest that neutral serine and threonine residues were more highly represented than many other uncharged (Q, N) and/or charged (R, H, D, E) polar residues. This finding reflects prior observations of a similar evolutionary preference for these small uncharged residues in eukaryotic HDPs (35). While the reason for this propensity is unknown, such residues may act as neutral “spacers” to aid incorporation of more biochemically reactive polar and charged residues within amphipathic HDPs. Also, given the availability of their hydroxyl moiety for H-bonding, serine and threonine residues may facilitate miscibility in aqueous vs. lipid environments (35, 36).

The current study also provided information regarding the global biophysical properties found within amphipathic bacteriocins. As similar studies have been carried out in eukaryotes (11), we were interested in whether the bacteriocin amphipathic domains differed substantively from those found in higher organisms with phylogenetically advanced immune systems, or whether key physicochemical parameters are essentially immutable (37–39). The bacteriocin sequences identified in the current study exhibited a net cationic charge, reflecting a property that is nearly universal in microbicidal HDPs of eukaryotes. Cationicity is thought to be important mechanism of selective HDP affinity for anionic membrane lipids (e.g., phosphatidylserine, cardiolipin and phosphatidylglycerol), which are enriched in prokaryotes, and inward rectifying net electronegative potential of many bacterial membranes (40–42). The bacteriocin sequences were moderately cationic with an average net charge of +1.1 (*n* = 308). By comparison, a parallel study using the same amphipathic search tool identified a somewhat higher net charge in eukaryotic HDPs (Q = +2.0; *n* = 907; 11). This difference in net charge was also reflected in the relative percentage of cationic residues within bacteriocin amphipathic spans (K+R = 8%) vs. those in eukaryotic HDPs (K+R = 16%). The biological reasons for the slightly lower charge density in bacteriocins are not known, but ostensibly could reflect the potential for a greater degree of compartmentalization of HDPs in eukaryotic cells, such that charged and potentially toxic microbicidal sequences are safely stored until targeted release.

Similarly, charge composition analyses revealed that of the cationic residues, lysine was preferred over arginine in the amphipathic spans of prokaryotic bacteriocins in the current study (K:R = 2:1), and in eukaryotes (K:R = 5:1) (11). Importantly, lysine and arginine residues interact with membrane phospholipid head groups in fundamentally different ways. The single ε-amino group of lysine can only form a monovalent hydrogen bond with one membrane phospholipid headgroup at a time. In contrast, the guanidinium amino moiety of arginine can form multiple hydrogen bonds with phospholipid headgroups simultaneously. These differences lead to alternate membrane perturbation events, with arginine generating negative Gaussian curvature (NGC) oriented to achieve both positive and negative curvature along two perpendicular directions, whereas lysine generates only negative curvature. These biophysical constraints are supported by studies that have found that lysine is less efficient at generating negative Gaussian curvature (NGC), and pore-like structures, than arginine (12–14, 23). Notably, many lysine-rich HDPs have a net hydrophobic propensity, a feature that may compensate for this reduced permeabilizing efficiency, in a phenomenon known as the “saddle-splay” rule (23).

The observed preference for lysine over arginine common in the amphipathic spans of HDPs of prokaryotic and eukaryotic organisms suggest a crucial biophysical constraint within α-helical HDPs enabling membrane permeabilization. Several concepts support this hypothesis, including: (1) lysine-rich domains may be more energetically favorable for the transition from random coil to α-helical structures, as is common among these peptides; (2) reduced arginine frequency may make amphipathic helices less toxic toward “self” [relative to prokaryote (e.g., bacteriocin) or eukaryote (e.g., defensin) host] membranes; (3) a specific K/R ratio may facilitate a interaction with a cognate receptor or lipid II/LPS, and avoid off-target effects on ion channels; and (4) this ratio may confer some alternate evolutionary advantage.

Lastly, the BACIIα formula and algorithm retrieved a large number of sequences it classified as bacteriocins, but are as yet uncharacterized. As a proof-of-concept, several prototypes of these unknown sequences prioritized based on logical selection criteria were synthesized and assessed for antimicrobial activity. Notably, each of these peptides exerted activity against a broad spectrum of human pathogens, with generally greater activity vs. Gram-negative pathogens. In addition, each of the peptides demonstrated differential activity in pH conditions simulation bloodstream vs. abscess / phagolysosomal contexts. Historically, bacteriocins have been generally viewed as having relatively narrow spectrum activity, and greatest potency against closely-related Gram-positive organisms. However, more recent studies show that bacteriocins have broad spectra, with microbicidal activity against Gram-negative and fungal organisms as well (43, 44). It is interesting that HDPs from a variety of prokaryotes and eukaryotes can be active against fungi. There are at least two plausible targets of HDPs in fungi: (1) fungal envelope and/or cell membrane; and (2) mitochondria, which in effect are considered ancestral prokaryotic endosymbionts. With respect to the former, mechanisms for HDP targeting of fungi are believed to be related to unique components such as sphingolipids, glycolipids, phosphatidic acid and ceramides (45, 46). Considerable data suggest HDPs may target specific proteins integral to the fungal surface (47, 48). With respect to mitochondria, it is known that certain eukaryotic HPDs such as Histatin-5 target energized fungal mitochondria (49). Moreover, our previous work has demonstrated that HDPs can induce regulated cell death mechanisms leading to fungal cell death (50). These latter reports are in alignment with our current findings.

In summary, development of the BACIIα search formula and algorithm allowed for high-dimensional and rapid screening of proteomic databases to discover putative new bacteriocin species. Moreover, this process enabled characterization of essential features of prokaryotic bacteriocins, revealing fundamental similarities and differences with respect to analogous eukaryotic HDPs. These results offer key insights into essential, immutable features, as well as plasticity of evolution of HDPs from prokaryotes and eukaryotes. In this regard, such knowledge should improve our understanding of host defense against infection, and provide important templates for development of innovative anti-infectives.

## Data Availability Statement

The raw data supporting the conclusions of this article will be made available by the authors, without undue reservation.

## Author Contributions

NY conceived of studies, performed computational analyses, and wrote the manuscript. DW wrote programs for data analysis. JA performed computational analyses and assisted with writing the manuscript. EL performed computational analyses for the manuscript. ML provided input and assisted with writing the manuscript. GW conceived of studies and wrote the manuscript. MY conceived of studies and wrote the manuscript. All authors contributed to the article and approved the submitted version.

## Conflict of Interest

The authors declare that the research was conducted in the absence of any commercial or financial relationships that could be construed as a potential conflict of interest.
